# The suspected unexpected and serious adverse events of antiretroviral drugs used as HIV prophylaxis in HIV uninfected persons

**DOI:** 10.7448/IAS.17.4.19733

**Published:** 2014-11-02

**Authors:** Ewa Pietraszkiewicz, Ewa Firlag-Burkacka, Andrzej Horban, Justyna D Kowalska

**Affiliations:** HIV Out-Patient Clinic, Hospital for Infectious Diseases, Warsaw, Poland

## Abstract

**Introduction:**

With increased usage of antiretroviral drugs (ARVs) in HIV uninfected persons proper reporting on suspected unexpected serious adverse reactions (SUSARs) and continued insight into serious adverse events (SAEs) is needed for adequate information on ARVs safety in such populations.

**Methods:**

We have evaluated medical documentation of persons receiving ARVs after non-occupationally HIV exposure (nPEP) during five concomitant years (2009–2013). SAEs and SUSARs were evaluated by two HIV physicians and defined according to international standards. In statistical methods, Kaplan Meier survival analysis was used to estimate the probability of SAE and Cox proportional hazard models to identify independent predictors of developing SAE. Only the first SAE was included in these analyses.

**Results:**

In total, 375 persons received nPEP. The most common reason was needle stick (43%), followed by unprotected sexual intercourse (17%), rape (10%) and first aid (10%). In 84 (22%) cases, the source patient was either known to be HIV positive or within a high risk group (active injecting drug user). In total, 170 SAEs were reported, 139 persons had only one SAE and majority developed it within first two weeks. The most frequent first SAEs were gastrointestinal disorders (22%), followed by general symptoms (9%), hypersensitivity reactions (1.6%) and CNS symptoms (1.3%). The remaining events were laboratory abnormalities of liver and kidney function, haematological disorders, other and unknown, each contributing to less than 1% of all patients. 8 (2.1%) patients have developed a SUSAR (bradycardia, vivid dreams, lymphadenopathy of the neck, increased platelet count, swelling and pain of large joints, swelling of lower limbs, peripheral oedema and loss of concentration). 22 (5.9%) persons discontinued nPEP due to adverse event and 19 (5.1%) required a paid sick leave from work. In multivariate analyzes, only age was independent predictor of developing SAE (HR 1.17; [95% CI 1.03–1.34]; p=0.02).

**Conclusions:**

In our observation, SAEs in reaction to nPEP were frequent yet usually mild events, mostly occurring in first two weeks and rarely causing discontinuation. The only significant factor increasing the risk of SAE was age. SUSARs were rare and moderately significant. More insight into this important area is required in order to ascertain proper pharmacovigilance of ARVs usage in HIV uninfected persons.

**Table 1:** Cox proportional hazard models for the risk of developing first SAE

		Univariate			Multivariate		
		
		Hazard ratio	95% CI	p	Hazard ratio	95% CI	p
Gender	Female	1.00			1.00		
	Male	0.9	0.64–1.26	0.53	1.05	0.73–1.50	0.81
Calendar year	2009						
	2010	0.79	0.47–1.34	0.38	0.76	0.44–1.31	0.33
	2011	0.90	0.51–1.60	0.72	0.86	0.47–1.56	0.62
	2012	1.24	0.78–1.97	0.36	1.11	0.67–1.84	0.68
	2013	1.52	0.95–2.43	0.080	1.17	0.63–2.16	0.62
Age	Per 5 years older	1.02	1.00–1.03	0.010	1.08	1.01–1.16	0.02
	Per 10 years older	1.18	1.04–1.35	0.010	1.17	1.03–1.34	0.02
NRTI	AZT/3TC	1.00			1.00		
	TDF/FTC	0.95	0.51/1.76	0.87	0.91	0.46–1.81	0.79
Regimen	2 ARVs	1.00			1.00		
	3 ARVs	1.33	0.95–1.86	0.097	1.22	0.75–1.99	0.41
Exposure risk	Sexual				1.00		
	Needle stick	1.10	0.74–1.63	0.64	0.98	0.62–1.54	0.92
	Other	0.82	0.52–1.29	0.38	0.80	0.49–1.32	0.39
Source patient HIV status	Unknown	1.00			1.00		
	HIV infected or high risk	0.85	0.56–1.29	0.45	0.83	0.51–1.33	0.44

